# The SITLESS project: exercise referral schemes enhanced by self-management strategies to battle sedentary behaviour in older adults: study protocol for a randomised controlled trial

**DOI:** 10.1186/s13063-017-1956-x

**Published:** 2017-05-18

**Authors:** Maria Giné-Garriga, Laura Coll-Planas, Míriam Guerra, Àlex Domingo, Marta Roqué, Paolo Caserotti, Michael Denkinger, Dietrich Rothenbacher, Mark A. Tully, Frank Kee, Emma McIntosh, Carme Martín-Borràs, Guillermo R. Oviedo, Javier Jerez-Roig, Marta Santiago, Oriol Sansano, Guillermo Varela, Mathias Skjødt, Katharina Wirth, Dhayana Dallmeier, Jochen Klenk, Jason J. Wilson, Nicole E. Blackburn, Manuela Deidda, Guillaume Lefebvre, Denise González, Antoni Salvà

**Affiliations:** 1 Facultat de Psicologia, Ciències de l’Educació i de l’Esport Blanquerna (Universitat Ramon Llull), C/ Císter 34, 08022 Barcelona, Spain; 2 Fundació Salut i Envelliment UAB, Casa Convalescència UAB, C/ Sant Antoni Maria Claret 171, 4a planta, 08041 Barcelona, Spain; 3 Syddansk Universitet, Campusvej 55, 5230 Odense M, Denmark; 4 Agaplesion Bethesda Clinic, Geriatric Research Unit Ulm University and Geriatric Center Ulm Alb-Donau, Zollernring 26, 89073 Ulm, Germany; 5 Institute of Epidemiology and Medical Biometry, Ulm University, 89081 Ulm, Germany; 6 UKCRC Centre of Excellence for Public Health (NI), Centre for Public Health, School of Medicine, Dentistry and Biomedical Sciences, Queen’s University Belfast, Belfast, UK; 7 Health Economics and Health Technology Assessment (HEHTA), Institute of Health and Wellbeing (IHW), University of Glasgow, Lilibank Gardens, Glasgow, G12 8RZ UK; 8 Sport Initiative et Loisir Bleu Association, BP 18104, 67038 Strasbourg, Cedex France; 9 Sport Initiative et Loisir Bleu Association, C/ Bailén 5 bajos, 08010 Barcelona, Spain

**Keywords:** Sedentary behaviour, Physical activity, Behaviour change, Older adults, Self-management strategies

## Abstract

**Background:**

Older adults are the fastest growing segment of the world‘s population. Recent evidence indicates that excessive sitting time is harmful to health, independent of meeting the recommended moderate to vigorous physical activity (PA) guidelines. The SITLESS project aims to determine whether exercise referral schemes (ERS) can be enhanced by self-management strategies (SMSs) to reduce sedentary behaviour (SB), increase PA and improve health, quality of life and function in the long term, as well as psychosocial outcomes in community-dwelling older European citizens from four countries, within a three-armed pragmatic randomised controlled trial, compared with ERS alone and also with general recommendations about PA.

**Methods:**

A total of 1338 older adults will be included in this study, recruited from four European countries through different existing primary prevention pathways. Participants will be randomly allocated into an ERS of 16 weeks (32 sessions, 45–60 min per session), ERS enhanced by seven sessions of SMSs and four telephone prompts, or a control group. Outcomes will be assessed at baseline, month 4 (end of ERS intervention), month 16 (12 months post intervention) and month 22 (18 months post intervention). Primary outcomes will include measures of SB (time spent sedentary) and PA (counts per minute). Secondary outcomes will include muscle and physical function, health economics’ related outcomes, anthropometry, quality of life, social networks, anxiety and depressive symptoms, disability, fear of falling, executive function and fatigue. A process evaluation will be conducted throughout the trial. The full analysis set will follow an intention-to-treat principle and will include all randomised participants for whom a baseline assessment is conducted. The study hypothesis will be tested with mixed linear models with repeated measures, to assess changes in the main outcomes (SB and PA) over time (baseline to month 22) and between study arms.

**Discussion:**

The findings of this study may help inform the design and implementation of more effective interventions to reduce SB and increase PA levels, and hence improve long-term health outcomes in the older adult population. SITLESS aims to support policy-makers in deciding how or whether ERS should be further implemented or restructured in order to increase its adherence, impact and cost-effectiveness.

**Trial registration:**

ClinicalTrials.gov, NCT02629666. Registered 19 November 2015.

**Electronic supplementary material:**

The online version of this article (doi:10.1186/s13063-017-1956-x) contains supplementary material, which is available to authorized users.

## Background

Older adults are the fastest growing segment of the world’s population [1]. Although prolonging life remains an important public health goal, of even greater significance is the preservation of functional and cognitive performance, and of the capacity to live independently during late life.

Being insufficiently active is associated with increased risk for major non-communicable diseases and all-cause mortality, which is related to increased healthcare costs [2–4]. It is estimated that people aged 65 years and over account for 30–40% of the total healthcare spend across Europe [5]. The burden of disease attributable to being insufficiently physically active has recently been estimated to be responsible for 6–9% of the total deaths worldwide and accounts for as many as 5.3 million deaths per year [2, 6]. In this context, it is estimated that across all ages, 31% of the global population do not meet current physical activity (PA) recommendations established by the World Health Organization (WHO) [7].

In the last decade, growing evidence indicates that excessive sitting time may be harmful to health, independent of meeting the recommended moderate-to-vigorous PA guidelines [8–13]. Time spent in sedentary behaviour (SB)—defined as any waking behaviour characterised by energy expenditure ≤ 1.5 Metabolic Equivalent Tasks (MET) while in a sitting or reclining posture [14]—has increased substantially over the last three decades [15]. Older adults are the most sedentary segment of society, as many spend over 75% of their waking day in SB [16]. Current evidence suggests that prolonged SB is associated with increased rates of several chronic diseases and all-cause mortality in older adults [17–20].

Primary care is a key setting for the promotion of PA. The most common model of PA promotion in primary care involves exercise referral schemes (ERS), whereby a general practitioner or another member of the primary care team (e.g. physiotherapist, nurse or pharmacist) identifies and refers insufficiently active individuals to a third-party service (often a sports centre or leisure facility) to conduct an exercise program [21, 22]. Despite recent studies showing the health benefits of such programs, most studies also demonstrate that such gains are rarely sustained [21]. Recent guidelines also advocate the use of established behavioural change techniques (BCTs) to promote increased levels of PA [23]. Among those, self-management strategies (SMSs) may successfully improve involvement in exercise in specific populations, increasing levels of daily PA, enhancing quality of life, improving mental health, producing greater confidence and increasing power to act [24].

Research focusing on interventions to reduce SB has only begun to emerge in the last five years and there have been few studies conducted in older adults. Recent studies targeting reduced SB in this population have included BCTs such as goal setting, instruction on how to perform the behaviour, self-monitoring and feedback of behaviour, prompts/cues and restructuring the physical environment [25–29]. Moderate quality evidence indicates that specific interventions have the potential to reduce SB in older adults [30]. These findings are supported by systematic reviews focusing on adults [31–33]. As far as we know, no previous studies have assessed the effects of an exercise-based intervention enhanced by SMSs addressing both increased daily PA and a reduction of SB in older adults.

Therefore, the SITLESS project aims to determine whether ERS can be enhanced by SMSs to reduce SB, increase PA and improve health, quality of life and function in the long term (22 months), as well as psychosocial outcomes in community-dwelling older European citizens from four countries, within a three-armed pragmatic randomised controlled trial (RCT), compared with ERS alone and also with general recommendations about PA. SITLESS will also collect health economics’ related outcomes, data on healthcare systems and community costs to perform cost-effectiveness analyses. SITLESS has the purpose to support policy-makers in deciding how or whether ERS should be further implemented or restructured in order to increase its adherence, impact and cost-effectiveness.

## Methods/design

### Study design

The present study protocol describes a multi-centre pragmatic three-armed parallel RCT. Outcomes will be assessed at baseline, month 4 (end of intervention), month 16 (12 months post intervention) and month 22 (18 months post intervention) which will be the main outcome assessment. The study protocol has been developed based on the Standard Protocol Items: Recommendations for Interventional Trials (SPIRIT) guidelines [34]. The study design was approved by the Ethics and Research Committee of each intervention site: The Ethics and Research Committee of Ramon Llull University (Fundació Blanquerna, Spain), The Regional Committees on Health Research Ethics for Southern Denmark (University of Southern Denmark, Denmark), Office for Research Ethics Committees in Northern Ireland (ORECNI) (Queen’s University of Belfast) and the Ethical Review Board of Ulm University (Ulm, Germany). Participation is voluntary and all participants will be asked to sign informed consent before the start of the study (see Additional file 1).

SITLESS, as a Responsible Research and Innovation project, has created guidance for the involvement of stakeholders in the project from the onset. Accordingly, four local advisory boards were created, one on each intervention site (Barcelona, Odense, Belfast, Ulm) and are periodically involved in the study. They comprise primary healthcare and sport professionals, older adults, policy-makers and other local stakeholders of relevance (e.g. health insurance, where relevant). A gender expert linked to the European project EGERA is part of the advisory board of Barcelona.

### Participants

A total of 1338 participants (randomised into three intervention groups of 446 participants) will be included in this study and recruited in four intervention sites (Barcelona, Odense, Belfast, Ulm). Each intervention site will be in charge of recruiting 335 participants and will collect relevant descriptive information regarding participants’ demographic characteristics (e.g. age, gender, marital status, living arrangements and educational background). Barcelona is the largest site of the study with 1,602,000 inhabitants in 2014, followed by Belfast with 333,871 at the 2015 census. Odense and Ulm have less than 200,000 inhabitants. Outcome measures will be also analysed to detect any between-site differences.

Participants are eligible if they are: (1) aged 65 years or above; (2) community-dwelling; (3) able to walk without the help of another person for at least 2 min with or without a walking aid; (4) have no major physical limitations as shown by a score on the Short Physical Performance Battery (SPPB) of 4 or above [35]; (5) insufficiently active as determined by the following screening question: ‘*Do you perform regular physical activity (PA) for at least 30 minutes five or more days of the week (referring only to PA that makes the participant become out of breath while doing it or such that it doesn’t allow him/her to maintain a conversation while doing the activity) (do not count regular walking)*’; and/or (6) report spending long periods of time in SB by answering affirmatively to the question: ‘*For most days, do you feel you sit for too long (6–8 hours or more a day)? Some examples might include when watching TV, working at the computer / laptop or when doing sitting-based hobbies such as sewing*’ [17]. Participants will be excluded if they: (1) have moderate or severe dementia when screened with the six-item screener to identify cognitive impairment, using a cutoff of three or more errors [36]; (2) have a medical condition which may interfere with the study design; (3) have unstable medical conditions (e.g. elevated blood pressure after medication, uncontrolled hypertension) or symptomatic cardiovascular diseases that contraindicates participation in PA; (4) expect not to be able to attend 75% of the ERS sessions throughout the intervention; and (5) have participated in an ERS in the six months prior to their entry into the study.

### Sample size

A sample size of 1338 participants (randomised to one of three groups of 446 participants) will be needed to detect a moderate effect size of 30 daily counts per minute (CPM) in a two-sided test, at a power of 80% and an α of 0.05, a common standard deviation of 139 of the mean and a 24% dropout rate. A change of 30 CPM is considered a measurable moderate effect size in this population, assessed with ActiGraph GT3X+, and 139 is the standard deviation for CPM found in the literature [37]. The present sample size allows estimation of overall efficacy.

Sample size computations were conducted with online software GRANMO (http://www.imim.es/ofertadeserveis/software-public/granmo/; last accessed on 21/7/2014).

### Study procedure

Recruitment strategies will be site-specific according to the different existing primary prevention pathways in each country. Primary care centres and other health-related centres of each intervention site will be informed and asked to participate. The research team will inform professionals about the background and the aims of the study. Health professionals who volunteer to participate will be further given all information regarding eligibility criteria and the recruitment strategy. Posters, flyers, newspapers, radio broadcasts and social media outlets will also be used to advertise the study and as additional recruitment strategies (Additional file 2: shows the SPIRIT checklist).

Health professionals or the local research team will assess interested individuals against the eligibility criteria. Eligible participants will have their name, gender, date of birth and phone number recorded, while individuals deemed ineligible or that do not want to participate will have their reason for declining as well as their gender and age recorded.

### Randomisation and blinding

During the first meeting, a researcher will explain the trial to interested individuals, give them an information sheet about the study and obtain their written informed consent (see Additional file 1). Participants will then be scheduled for the baseline testing session. After the baseline assessment, each participant will be centrally randomised to one of the three study groups (e.g. ERS + SMS, ERS or the control group), using a computer-based random-block randomisation scheme, clustering by couples when cohabitees wish to be enrolled together. Concealed randomisation will be conducted centrally at FSiE, after each participant has been included in the study, assigned an identification code and has completed the study baseline assessment.

The trial has an open design with blind assessment of outcomes. Researchers conducting the baseline assessments will be blind to group allocation. The statistician will also be blind to group allocation until completion of the statistical analysis. Participants will be asked not to reveal group allocation when undergoing follow-up measurements, as researchers conducting follow-up measurements will be blind to group allocation. To assess the extent to which blinding has been preserved, researchers will record the number of cases in which allocation was revealed.

### Study interventions

A complex intervention [38] consisting of a PA program with self-management strategies (ERS + SMS) will be delivered in primary care or community settings in urban areas of Barcelona (Spain), Odense (Denmark), Belfast (UK) and Ulm (Germany) (see Table 1). All trainers in charge of conducting both intervention groups will undergo a standardised training.

**Table 1:** General information of the ERS and SMS interventions

Name of the program	Program components	Training responsible	Duration	General structure of each session
ERS intervention	Aerobic training. Strength-based/ endurance exercises. Balance-based functional exercises. Flexibility exercises.	Specially trained PA specialist: physical therapist; sport professional/trainer; ergotherapist with specific health qualification. Sessions will be always performed under the supervision of the same trainer.	16 weeks. Two sessions per week of 45–60 min. Ask each participant to perform a third session on their own such as a 30-min walk. The intervention will be conducted in an indoor primary-care or sports facility. Municipality facilities (e.g. activity centres for older adults).	All training sessions will begin with a 5–10 min warm-up focusing on social and physical interactions. Followed by 35 min of different exercises adapted to each individual’s functional level (according to the participants’ SPPB score^a^). All training sessions will end with cool-down (breathing exercises and stretching for 5–10 min.
SMS intervention	Raising awareness on differences, associations, risks and benefits of SB and PA. Setting personal activity goals (long-term achievement goals). Enhancing motivation. Goal-setting focusing separately on SB and PA. Self-monitoring (pedometer and activity diary). External monitoring (instructor). Problem-solving according to the IDEA.^b^ Social influence and social support. Raising awareness on facilitators and barriers of PA and SB at home and neighbourhood. Environmental signposting.	The same specialist for the ERS intervention.	Total of 30 weeks. 1 one-to-one session (week 1; 40 min). 6 group-based sessions (weeks 3, 4, 5, 7, 9 and 11; 45–60 min). 4 telephone calls (weeks 15, 20, 25 and 30; 20 min).	The SMS sessions include the following activities: introducing the project to the participant, developing a rapport, setting a meaningful long-term goal to be achieved at the end of the intervention, identifying facilitators and barriers of PA and SB at home and neighbourhood in a group dynamic, environmental signposting to help engaging participants in local opportunities to do PA, checking daily step counts registered in the activity diary and setting individual goals to increase steps or other physical activities, setting individual goals to reduce siting time set choosing recommendations (SITLESS tips) for decreasing SB, problem-solving techniques to overcome barriers to being less sedentary and more active according to the IDEA^b^ problem-solving.

^a^Total SPPB score ranges from 0 (worst performance) to 12 (best performance); participants will be classified into three different functional performance levels according to the results obtained: low = 4–6; medium = 7–9; high functional level = 10–12 points

^b^ *IDEA* Identifying the problem, Develop a list of solutions, Evaluate the solutions and Analyse how the plan worked

#### Physical activity intervention (ERS)

Participants will undergo a 16-week PA program, consisting of two sessions per week (45–60 min each session). Participants will be asked to perform the activity at a moderate-to-vigorous intensity (according to each individual’s fitness levels) during the main part of each session. Intensity will be estimated using the modified Borg Scale of Perceived Exertion [39] (e.g. moderate-intensity activity will be considered 4–6 and vigorous-intensity activity will be 7–9) or with training loads (i.e. ankle weights and dumbbells) corresponding to 70–80% of one repetition maximum, adjusted progressively during the training period.

The ERS program will be based on a combination of aerobic, strength-based and balance activities. Aerobic training may include walking, rowing, using an elliptical trainer, cycling, fitness/water aerobics, Nordic walking, swimming, dancing and/or any other activity such as games which increase heart rate and respiratory frequency. During the first week, these activities will be performed at a moderate intensity, with bout duration based on each individual aerobic level. Progression will include increasing the duration and/or the intensity (e.g. increasing resistance on the bike ergometer or increasing walking speed on Nordic walking) up to a maximum of 9 of the same Borg Scale. The intervention will be tailored for the participants with a low functional level (score of 4–6 of the SPPB), in which more strength training will be performed.

Lower-body strength exercises will include functional tasks such as rising from a chair, stair climbing, knee bends, floor transfer, walking, lunges, leg squat, leg extension, leg flexion, calf raise, lower back and abdominal curl using ankle weights, elastic bands, training devices (e.g. leg press), free weights or any other material available (e.g. water bottles or sand sacks). Upper-body exercises may include pulldowns, low row, chest press, shoulder press and biceps curl. The first session will be used to calculate the baseline intensity. The first two weeks of the intervention will be used as familiarisation, with the focus on technique of the different exercises. Following the two-week period, training intensity will be individually calculated by the 4–8 repetition maximum method (i.e. maximum number of repetitions to failure between 4 and 8) for each exercise using different training tools devices (e.g. fixed weights machine, free weights, elastic bands) and body weight (e.g. loaded sit-to-stand, squats, lunges). Lower-body exercises will be performed explosively (e.g. as rapid as possible) during the concentric phase of the movement and controlled during the eccentric phase.

Balance-based exercises will focus on functional activities. Balance activities will be designed to challenge the visual (e.g. eyes open/closed), vestibular (e.g. move head) and somatosensory (e.g. stand on foam) systems. Static balance will consist of two-leg and one-leg balance with toes or heels raised and tandem standing with eyes open or closed on different surfaces. When training dynamic balance, activities such as walking on different surfaces, with varied elevations, and performing a dual task (cognitive and functional task such as catching, throwing and reaching), incorporating different gait patterns (e.g. narrow walking, longer strides, zigzag walking) and variations in gait speed, will be performed. Balance exercises will include function-focused activities such as walking with obstacles while wearing standard sunglasses (worn over corrective lenses as needed) to mimic a semi-dark environment, walking while carrying a package that obstructed the view of the feet and walking while picking up objects from the floor. Initially the participants will perform one or two sets of 6–8 repetitions of each exercise; the number of repetitions will be increased when a participant is able to complete eight repetitions showing no difficulty; the maximum number of repetitions will be 20. Three sets of exercises of increasing complexity will be designed; when an easier step will be achieved without assistance, the individual will be asked to perform the next more complex set of exercises.

Participants will be encouraged to report any negative sign or symptom resulting from the exercises during the sessions. A brief summary on the ERS and SMSs interventions can be found in Table 1.

#### ERS + SMS sessions

In order to fully inform the development of the SMSs intervention, a number of preliminary steps were carried out. A systematic review of interventions which aimed to reduce SB in older adults was undertaken to identify the components of these interventions, particularly BCTs. Once these were established, each intervention site conducted focus groups with older adults in order to get their opinions on the proposed BCTs which would be utilised in the SMSs intervention. On completion of both these tasks, and guided by social-cognitive theory, an initial logic model was developed to help determine how different intervention components (i.e. inputs) might have an impact on the proposed study outcomes (e.g. SB, PA, quality of life and functional capacity). The initial SMSs intervention was tested by each intervention site as part of a feasibility study before it was refined for the final SITLESS trial. SMSs training materials were developed and workshops were conducted by each partner to help fully explain the SMSs intervention to the instructors. Monitoring and support for the SMSs instructors is planned on a monthly basis using a mixture of face-to-face meetings and teleconferences.

Participants randomised to this group will take part in a local ERS combined with SMS intervention lasting 30 weeks in total. Both the ERS and SMS sessions will be led by suitably qualified fitness instructors who have been previously trained regarding the SMS intervention. There will be 11 SMS sessions: one taking place before the ERS intervention, seven during the ERS intervention and three after the ERS. BCTs will be used to guide the structure and content of the sessions [40]. These sessions will consist of a one-to-one session, six group-based sessions in local leisure / community centres and four telephone calls to offer additional support to the participants and to find how they are getting on with the SMS intervention. The SMS sessions will be structured as listed below.

One-to-one visit (Familiarisation Stage): the main aims of the one-to-one visit will be to introduce the SMSs intervention and materials to the participant and to start developing a rapport. The participant will be given an information booklet which will give further details on SB and some ideas on how to sit less and be more active (i.e. SITLESS tips). The participant will also be given a Yamax DigiWalker SW-200 pedometer to wear during the course of the SMSs intervention and will be shown how to put this on by the instructor (the participant information booklet will contain instructions on how to use the pedometer). The participant will be given an activity diary to use during the duration of the SMSs intervention to monitor their daily step counts, weekly time in PA / exercise and use of SITLESS tips. This will also allow them to monitor their daily step counts and weekly time in PA / exercise during the first three weeks of the intervention in order to develop baseline readings before the first group-based session. The final task will be to establish long-term functional goals that the participant would like to achieve in the months after completing the intervention. The one-to-one visit is expected to last approximately 50 min.

Six group-based sessions in the local leisure or community centre (Ramping and Maintenance Stages): the main aims of the group-based sessions will be to retrieve the participants’ step counts and weekly time in PA / exercise recorded in their activity diaries and their use of SITLESS tips as well as agreeing on participants’ goals for the following weeks. The participants will be encouraged to gradually increase their daily step counts, weekly time in PA / exercise and their use of the SITLESS tips. Each group-based session will also cover a specific theme such as goal-setting, identifying barriers and facilitators for PA and SB in the neighbourhood and at home, environmental signposting and problem-solving techniques. It is hoped that these sessions will act as an opportunity to improve participants’ motivation and increase the level of social support to sitting less and being more active. Each group-based session is expected to last approximately 45–60 min.

Four telephone calls (Adherence Stage): the main aims of the telephone calls are to provide motivational support and advice to participants to sit less and be more active as well as trying to understand and support participants to maintain their goals (daily step counts, weekly time in PA / exercise and SITLESS tips). Each telephone call is expected to last no more than 20 min.

#### Control group (CG)

Researchers will give to all participants during the first informative meeting (prior assessment) a written general booklet standardised across sites with the WHO recommendation regarding PA regular practice for health. During the intervention, a health advice meeting with standardised topics about healthy lifestyle and feedback on some outcomes will be held twice in the Primary Health Centre (at week 5 and at week 11). Researchers will send a letter or make a phone call prior to the next assessment.

### Outcome assessment

**Fig. 1:**
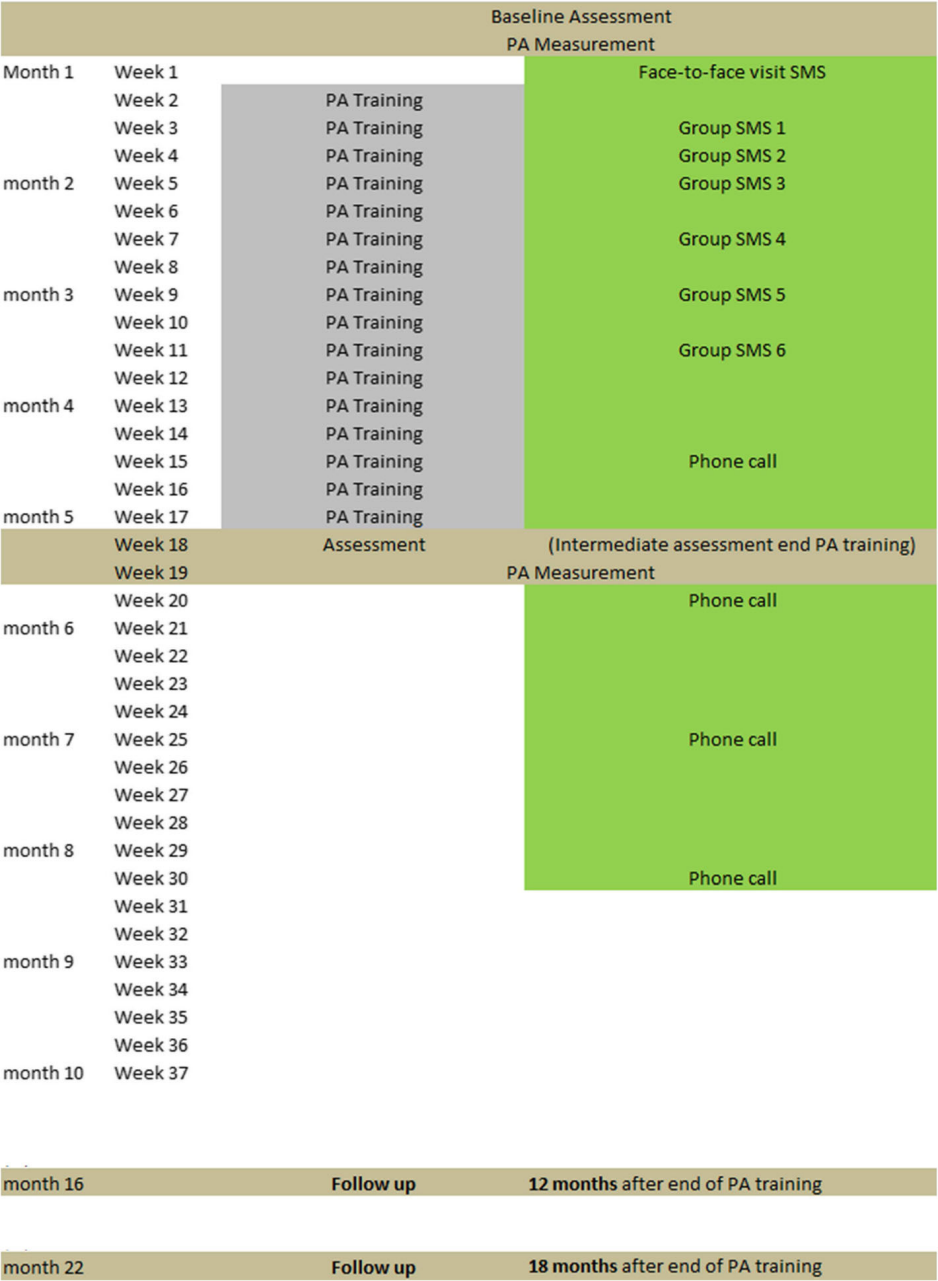
Schedule of SITLESS interventions

**Fig. 2:**
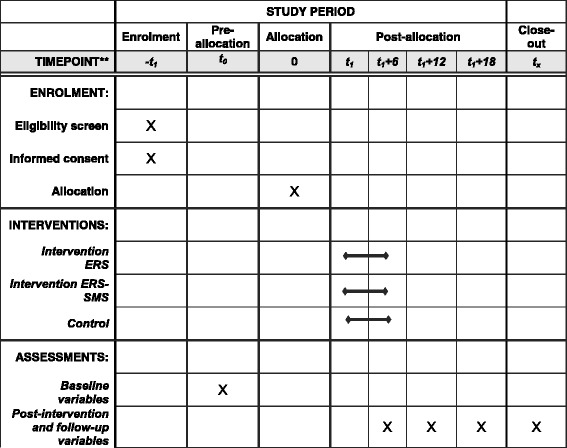
SPIRIT figure

The ERS group will take 16 weeks and the ERS + SMS group will take 30 weeks (including the four phone calls) (see Fig. 1). Assessments will be conducted by the same assessors at the following time points: T0 = baseline pre-intervention, T1 = at month 4 post intervention, T2 = at month 16 (12 months after the end of the intervention), and T3 = at month 22 (18 months after the end of the intervention). (Fig. 2 shows the SPIRIT figure). All researchers in charge of conducting the assessments will undergo a standardised training session.

Personal information regarding age, gender, marital status, living arrangement, educational background, medical conditions, and smoking and alcohol habits will be collected at baseline.

The costs of the interventions (ERS, SMS and the CG intervention) will be also collected by a structured questionnaire.

Primary outcomes of the study include: (1) SB as sitting time and the number of minutes spent in activities requiring ≤ 1.5 MET, as objectively measured by hip worn ActiGraph® accelerometer, and in addition with ActivPal® (used in Barcelona and Ulm) and Axivity® (used in Odense and Belfast) accelerometers worn on the thigh, and as self-reported according to the Patient-centered Assessment and Counseling for Exercise (PACE), Sedentary Behavior Questionnaire); and (2) physical activity as daily CPM and intensity of exercise performed, as measured by ActiGraph®. Primary and secondary measures will be collected at the four time points. For an overview of primary and secondary outcomes, outcome measures, instruments and assessment time points, see Table 2.

**Table 2:** Overview of outcomes, outcome measures, instruments and assessment time points

Outcomes	Outcome measures	Instrument	Assessment time point^a^
Personal information	Age, gender, civil status, living arrangement, educational background, medical conditions, and smoking and alcohol habits.	Primary care records Self-report	T0
Primary outcomes			
Sedentary behaviour	Sitting time and the number of minutes spent in activities requiring ≤ 1.5 Metabolic Equivalent Tasks.	Actigraph® ActivPal® Axivity® Sedentary Behavior Questionnaire	T0, T1, T2, T3
Physical activity	Daily counts per minute and intensity of exercise, and daily step counts.	Actigraph®	T0, T1, T2, T3
Secondary outcomes			
Physical function	General function Aerobic capacity Static balance	SPPB 2-minutes’ walk test Unipedal stance	T0, T1, T2, T3
Muscle function	Handgrip strength	Takei analogue Hand Grip Dynamometer	T0, T1, T2, T3
	Mean strength and power with concentric contraction of isoinertial movement performing 3 exercises: (a) 30-s chair stand rise; (b) five repetitions of arm curl with both hands using a 2-kg and 4-kg weight; and (c) four counter-movement jumps.	Linear encoder	
Health economics’ related outcomes	Use of sport services, and use of health and social services, medications, number of falls.	Interview	T0, T1, T2, T3
Anthropometry	Weight, height, body mass index, waist and hip circumference.		T0, T1, T2, T3
Bioimpedance	% fat; % muscle	Tanita BC 420S MA bioimpedance analyser	T0, T1, T2, T3
Blood pressure	Systolic and diastolic blood pressure; heart rate.	OMRON M6 comfort	T0, T1, T2, T3
Activities of Daily Living		6-item questionnaire	T0, T1, T2, T3
Self-rated health and health-related quality of life		SF-12 EUROQOL-5D ICECAP-O	T0, T1, T2, T3
Anxiety		HADS	T0, T1, T2, T3
Depressive symptoms		HADS	T0, T1, T2, T3
Social network		Lubben Social Network Scale-6	T0, T1, T2, T3
Physical activity self-regulation		12-item Physical Activity Self-Regulation Scale	T0, T1, T2, T3
Self-efficacy for exercise		Marcus’s Self-Efficacy Questionnaire	T0, T1, T2, T3
Disability		Short form Late Life Function and Disability Index	T0, T1, T2, T3
Fear of falling		Short Falls Efficacy Scale – International	T0, T1, T2, T3
Loneliness		Short form De Jong Gierveld Loneliness Scale	T0, T1, T2, T3
Executive function		Trail Making Test	T0, T1, T2, T3
Physical fatigue		Pittsburg Fatigability Scale	T0, T1, T2, T3
In a subsample:			
Level of frailty-associated biomarkers and inflammation	IL-6, hsCRP, TNF-alpha, IGF-1.	Blood sample	T0, T1
Sarcopenia-associated markers of muscle quality	Myostatin, IL-6, IL-8, IL-15, VEGF, BDNF, FGF21, irisin, myostatin, Type 2/Type 1 fibre ratio, Wnt and Notch signaling, CDC42	Muscle biopsy	T1

^a^Assessment time points: T0 = baseline pre-intervention, T1 = at month 4 post intervention, T2 = at month 16 (12 months after the end of the intervention), and T3 = at month 22 (18 months after the end of the intervention)

*SPPB* Short Physical Performance Battery, *ICECAP-O* ICEpop CAPability measure for Older people, *HADS* Hospital Anxiety and Depression Scale

### Economic evaluation

The health economic evaluation performed alongside the SITLESS clinical trial will be integral to the main RCT, providing useful guidance upon the cost-effectiveness of the intervention. It will be conducted following NICE [41] recent economics method guidance for the implementation of the cost-effectiveness analysis, as well as CHEERS guidelines for the reporting of the results [42, 43].

Being a population health intervention conducted in a multi-country setting, the health economic evaluation of the SITLESS intervention poses specific additional challenges related to the evaluation of complex public health interventions of this type [44] as well as to the multi-country nature of the intervention.

The costs of providing the ERS enhanced by SMSs (the ‘intervention’ costs) will be identified from a national health service/publicly funded and personal social services perspective alongside potential cost impacts (costs incurred as well as cost savings) including hospitalisations, accident and emergency visits, appointments with health professionals (health visitor, general practitioner contacts) as well as personal social services (i.e. social services and community care).

An economic logic model will be developed to systematically identify all relevant costs and cost savings as well as to identify potential longer term cost impacts. Following a multi-country costing approach, costs will be evaluated using country-specific unit cost estimates weights; furthermore, costs will be translated into a common currency by using the PPP statistic reported by OECD. Unit cost data will be identified from routine country-specific sources: NHS Reference Costs, PSSRU (UK); InEK - Institute for the Hospital Remuneration System (Germany); Health Department of Catalonia (Spain); Danish National Health Register (Denmark) in order to use country-specific price weights. Furthermore, cross-country comparability between data sources will be checked.

As for the outcomes, self-reported health-related quality-adjusted life-years (QALYs) will be obtained using the EQ-5D-5 L EuroQol [45, 46] instrument as well as the newly developed capabilities measure of outcomes for older people, the ICECAP-O instrument [47]. The multinational aspect of the analysis requires the use of country-specific tariffs, which reflect country-specific differences in health perceptions and preferences and might severely affect Cost-Utility analysis [48–50]. When available, country-specific tariffs will be used to evaluate health outcomes.

The within trial analysis will explicitly take the multi-country nature of the SITLESS trial into account, by using appropriate methodologies as suggested by the most recent literature and best practices. The multi-national feature of the SITLESS trial implies a hierarchical structure of the data and unobserved heterogeneity between clusters that needs to be adequately modelled with appropriate statistical and econometric techniques [51, 52]. Following best practice approaches [53–55] a multiple imputation procedure using chained equations (MICE) will be used to impute missing data separately for each arm of the trial.

Within-trial results will be reported and presented as an incremental cost-utility ratio with the joint distribution of cost/utility pairs being represented on the cost-effectiveness plane and with a cost-effectiveness acceptability curve (CEAC) [56].

The within-trial economic evaluation results will be combined with evidence from the literature linking short-term outcomes with longer-term outcomes to produce a long-term cost-effectiveness decision analytic model. A Markov model will be the envisaged analytic tool to evaluate population health interventions and to examine outcomes over an extended period of time and it will be developed alongside Frew et al. [57] and Roux et al. [58]. However, given that such long-term impacts are likely to be reliant on some untestable assumptions, the model will comprise a detailed sensitivity analysis to explore how cost-effectiveness will vary within all realistic ranges of costs and outcomes.

As recommended by the UK’s NICE guidance on economic evaluation of population health interventions an annual population health discount rate of 1.5% will be applied to costs and outcomes.

### Ancillary blood and muscle sub-studies

Two sub-studies will be performed to further elucidate possible mechanistic pathways between SB, PA and our primary and secondary outcomes. Biomarkers will be assessed at baseline and after intervention in blood samples and only after the intervention in muscle biopsies.

#### Subproject A

Subproject A is an analysis of changes in established core blood biomarkers associated with the intervention. This exploratory sub-study will analyse blood samples from 456 participants (95 participants per arm from two interventional sites n = 570, considering 20% loss of follow-up n = 456). Blood will be drawn from participants at baseline (T0) and at T1 to explore whether the intervention modifies established and accessible blood biomarkers (IL-6, hsCRP, TNF-alpha, IGF-1, WBC, RBC, platelets). Irrespective of the study arm, all participants with an improvement in PA will be analysed and compared to those without improvement in PA.

#### Subproject B

Subproject B is an analysis of post-intervention effects on muscle fibres or muscle tissue components in older adults. This exploratory sub-study will analyse muscle tissue from 60 participants to generate hypotheses on potential effects of reducing SB on muscle tissue. The muscle biopsy will be performed at week 21 (after assessing the post-intervention activity level with accelerometers) from a biopsy specialist and biomarkers will be assessed (Myostatin, IL-8, IL-15, VEGF, BDNF, FGF21, irisin, Type 2/Type 1 fibre ratio, Wnt and Notch signalling, CDC42). In this exploratory analysis, the physical activity profile or the sedentary profile from baseline assessment and of the post-intervention period, respectively, will be used as a main exposure marker to be analysed in relationship to the various biomarker measurements. Beside descriptive and summary measures we will use a multivariable analysis approach to quantify the relationship, and simultaneously adjusting for potential confounders such as age, gender and co-morbidities.

### Process evaluation

SITLESS follows the guidance on ‘Process evaluation of complex interventions’ from the Medical Research Council (MRC) for conducting the process evaluation of the study [59]. It was applied in the feasibility study to understand the feasibility of the recruitment strategy and of the SMS intervention and, hence, optimise its design and evaluation for the full trial.

The process evaluation of the full trial will be coordinated by a non-intervention site and is aimed at providing greater confidence in conclusions about effectiveness of the intervention by assessing: (1) the quantity and quality of what was delivered (implementation) regarding the PA and the SMSs interventions; and (2) the generalisability by understanding the role of context. Moreover, the process evaluation will help to understand the mechanisms of impact.

All intervention sites will combine their findings to gain a greater understanding of how the SMSs and the PA interventions are implemented and the extent to which they work. Methodologically, basic quantitative measures of implementation such as fidelity and adherence will be combined with qualitative data, including observation on sessions, focus groups and interviews with a purposeful sample of participants with different profiles. Moreover, the sessions on monitoring and support of the SMSs instructors will be used to gather further information on implementation challenges. Research teams will provide context information on each intervention site.

Qualitative and quantitative findings will be triangulated according to a standardised protocol that will entail: (1) sorting themes and following threads; and, where relevant, (2) seeking convergence, complementarity, silence and dissonance in themes, looking for either full or partial agreement or disagreement across the methods [60]. Our methods will follow best practice reporting guidelines for qualitative studies [61].

### Data and statistical analysis

The full analysis set will follow the intention-to-treat principle and will include all randomised participants for whom a baseline assessment is conducted, regardless of later temporary or permanent loss to follow-up, withdrawal or drop-out of the study.

The study hypothesis will be tested with mixed linear models with repeated measures, to assess changes in main outcomes (SB and CPM) over time (T0 to T3) and between-study arms. A diagonal matrix design will be considered for the within-participants covariance matrices (covariance structure for repeated measures factors). Fixed effects in the analyses will be treatment group and country (study site), the latter based on its relevance as proxy for intervention and cultural differences. Random effects may be considered for the ERS + SMS group and ERS group, to take into account within-study clustering. A diagonal matrix design will be considered for the between-participants covariance matrices (covariance structure for random factors). The between-participants correlations induced by randomisation by centre and the clustering of cohabiting individuals will be taken into account in the models. Further testing of the study hypotheses will be conducted through covariance analyses, including in the model relevant covariates (age, gender, BMI, SPPB level and frailty), and effect modifiers.

Secondary analyses will be conducted to explore changes over time in the relative efficacy of the interventions. Mixed linear models with repeated measures will be built to assess changes over time between intervention groups in main and secondary variables, at baseline (T0) and T1, T2 and T3. Interaction terms between treatment and time will be considered to test for differences over time between treatments.

A sensitivity analysis will be conducted, restricted to the ‘per protocol’ dataset, comprising all participants with no protocol violations and complete (e.g. no missing data) measurements for the primary variables for all study assessments (baseline, end of treatment, first follow-up, second follow-up). A second sensitivity analyses will be conducted for any primary variable with a substantial number of missing values (higher than 5%), imputing missing data by the ‘last observation carried forward’ method.

No subgroup analyses are planned in advance. Any subgroup analysis conducted will be treated with caution and output will be treated as exploratory rather than definitive. Missing data will be imputed through a multiple imputation approach, imputing missing values with the corresponding average values from the set of participants in the control group in the same country as the participant, matched for age, gender, baseline PA and baseline SPPB score.

Two-sided tests of statistical significance will be used in all statistical analyses. Estimates of the size of treatment effects will be presented together with confidence intervals, in addition to significance tests. Significance levels will be set to a 5% level, applying the Bonferroni correction to adjust for type I error criteria. All analyses will be performed with SPSS version 20 or Stata version 12.

## Discussion

This study provides a protocol for a multicentre pragmatic RCT to assess the effectiveness of existing ERS enhanced by SMSs to reduce SB, increase PA and improve health, quality of life, function and psychosocial outcomes in the long term (22 months) in older adults included in four European countries.

Currently, numerous RCTs have assessed the effects of PA-based interventions to improve health outcomes [21]. However, interventions aimed at reducing SB in older adults have only begun to emerge. There is growing evidence on how to promote healthier habits through SMSs based on behavioural interventions, but the integration of this knowledge into one complex and pragmatic RCT is a novel approach, as well as the expected impact in the long term. The foundation of this study is the existing ERS, which are implemented across Europe, but the adherence of professionals and older adults to ERS appears to be relatively poor. Therefore, SITLESS aims to impact health policy-makers with evidence-based knowledge to improve existing ERS by making them more effective.

Several national and international public health guidelines explicitly recommend that older adults should reduce their sedentary time and break prolonged periods of sitting to promote healthy ageing and wellbeing [62]. However, to date, few studies have investigated determinants of sitting and barriers and facilitators to change SB in older adults [63, 64]. Studies have reported determinants, barriers and facilitators specific to sitting that are different from those for PA. This might be due to the unique nature of SB, which occurs continuously during the day, compared to finite and set periods of PA or exercise occurring during the week. This suggests that interventions to reduce SB need to be specifically integrated into daily life.

When designing interventions to reduce SB and increase PA levels, rather than solely focusing on activities of at least moderate intensity, there is emerging evidence that replacing sitting time with standing or light PA may also provide substantial public health benefits [10, 65]. Some researchers had recognised that many older adults find it difficult to meet the PA guidelines of ≥150 min of moderate PA per week [66]. Therefore, a theoretic advantage of the SITLESS study is that promoting light PA may maximise the likelihood of people increasing their volume of PA along the continuum to a higher PA level [67].

Older adults are often unaware of exactly how long, where, when and why they sit [64]. Comparisons between self-reported and objective measures of SB have shown that older adults underestimate the time they spend sitting [16]. In the SITLESS trial, a combination of self-report and objective measures will be used. Moreover, the intervention itself pretends rising awareness on SB among participants.

The findings of this study will help inform the design of more effective interventions to reduce SB and increase PA levels, and hence improve health outcomes in the older adult population in the long term. The results of the present study may help to assist policy-makers of different European countries in deciding how or whether this intervention should be implemented widely or restructured.

### Study limitations

The SITLESS study faces an important challenge on how to standardise ERS and SMSs interventions across four European countries with different healthcare pathways. However, this is the first large RCT comparing ERS and ERS enhanced with SMSs to reduce SB in older adults, assessing many validated and objective outcome measures. SITLESS incorporates a process evaluation procedure throughout the trial providing greater confidence in conclusions about effectiveness.

### Trial status

This initial phase of this project commenced in May 2015. A feasibility study was performed from December 2015 until June 2016. The recruitment for the trial started in July 2016. At the time of submission of this protocol, less than 250 participants have been included in the study over a six-month recruitment period. To date, none of the participants have completed the intervention and no adverse events have been reported.

## Additional files

Additional file 1: Participant information and informed consent. (PDF 257 kb)

Additional file 2: SPIRIT checklist. (DOC 120 kb)

## Abbreviations

BCTs: Behaviour change techniques
CG: Control group
CPM: Counts per minute
ERS: Exercise referral schemes
HADS: Hospital Anxiety and Depression Scale
ICECAP-O: ICEpop CAPability measure for Older people
MET: Metabolic Equivalent Tasks
MRC: Medical Research Council
PA: Physical activity
PACE: Patient-centered Assessment and Counseling for Exercise
SB: Sedentary behaviour
SMSs: Self-management strategies
SPPB: Short Physical Performance Battery
WHO: World Health Organization
