# Supplementary material for: The SITLESS project: exercise referral schemes enhanced by self-management strategies to battle sedentary behaviour in older adults: study protocol for a randomised controlled trial
Source: Trials. 2017 May 18;18:221. doi: 10.1186/s13063-017-1956-x (PMC5437412; doi:10.1186/s13063-017-1956-x)
Supplement: Supplementary file 1 — Participant information and informed consent. (PDF 257 kb) [file 13063_2017_1956_MOESM1_ESM.pdf]

Title of the project. SitLESS: Exercise Referral Schemes enhanced by Self-management Strategies to battle sedentary behaviour.

Dear participant,

Thank you for your interest in the study entitled **Exercise Referral Schemes enhanced by Self-management Strategies to battle sedentary behaviour.**

Aim of the study: To evaluate the effectiveness of an intervention in the long term which combines a physical activity program with behaviour change strategies to reduce sedentary behaviour and improve physical function.

Voluntary participation: Your participation is totally voluntary. You are free to participate on this study and whether you decide to participle or not, your decision will not affect your health care attention or the relationship that you may have with your health care professionals.

Study procedures: We will request the collaboration of people over 65 years who live in community settings, who are able to walk independently, with no important physical limitations, and who have a low physical activity level (insufficiently active). We will follow a protocol designed by the research team, which has been supervised by a scientific and ethics committee. We will carry out a four-month physical activity program, with sessions held twice a week (a total of 32 sessions) in which we will especially perform functional exercises including aerobic, balance and strength activities. Sessions have been especially designed for older adults, considering their individual characteristics and the progression of each participant.

We will also conduct 6 individual sessions to find the best behaviour change strategy for each participant.

During the first visit, we will explain you the study procedure and answer all your questions and doubts. We will then ask each of you to sign the informed consent.

We will also interview you in order to go through some questionnaires including sociodemographic data, health-related quality of life, incidence of depressive symptoms, and physical activity level. We will also assess physical function through non-invasive functional tests. To measure sedentary behaviour we will ask you to wear a small instrument with a belt on your hip during the first week of the study. This device will record the following information: weekly caloric expenditure, daily number of steps and hours of sedentary activity during this period.
In the first appointment, we will inform you about which intervention you will do.

Confidentiality: All data collected on your participation will be treated confidentially. In the work lists, there will only appear the code which will be assigned to you for the study. In the final report or in the event of communicating these results to the scientific community, your identity will stay anonymous. Some medical information will be collected through the health history available in the Primary Care Center.

Responsibility of the study: The research team assume the responsibility of the study. If you would like to ask any question related to the study or if you need help for any health issue related to the study, please, do not hesitate to contact:

[Each partner should decide their contact procedure]

We really appreciate your willingness to collaborate with us on this study.

Informed consent to participate in the study

Title: SitLESS: Exercise Referral Schemes enhanced by Self-management Strategies to battle sedentary behaviour
Center: [indicate center]
Responsible researcher:
Address:
Phone number:

Mark all boxes:

- As a participant on this study, I confirm that I have read all the information, as well as the informed consent. ☐

- I understand that I have no obligation to participate in this study. I can withdraw the study at any moment and my decision will not have any unfavourable consequences on me, and neither will it affect any service I receive. ☐

- I understand that the obtained information will be entered into a database for its posterior analysis and the information will be treated in strict confidence by researchers. Furthermore, these data will not be used by other persons or other purposes. ☐

- I understand that I will not be identified in the final report, ensuring confidentiality. ☐

- I confirm that I have understood all the information and I agree to participate in the study. ☐

………………………………………………	………………………	……………………..
Participant's name	Signature	Date

Participant

Title: SitLESS: Exercise Referral Schemes enhanced by Self-management Strategies to battle sedentary behaviour
Center: [indicate center]
Responsible researcher:
Address:
Phone number:

Mark all boxes:

- As a participant on this study, I confirm that I have read all the information, as well as the informed consent. ☐

- I understand that I have no obligation to participate in this study. I can withdraw the study at any moment and my decision will not have any unfavourable consequences on me, and neither will it affect any service I receive. ☐

- I understand that the obtained information will be entered into a database for its posterior analysis and the information will be treated in strict confidence by researchers. Furthermore, these data will not be used by other persons or other purposes ☐

- I understand that I will not be identified in the final report, ensuring confidentiality. ☐

- I confirm that I have understood all the information and I agree to participate in the study. ☐

……………………………………………	………………………	…………………….
Participant's name	Signature	Date

Professional
